# The National Publishing Company

**Published:** 1867-01

**Authors:** 


The National Publishing Company will publish a work by Hon. Alexander H. Stephens, entitled "A History of the Late War between the States—tracing its origin, causes and results." This work will appear at an early date.

